# Daytime Restricted Feeding Modifies the Temporal Expression of *CYP1A1* and Attenuated Damage Induced by Benzo[a]pyrene in Rat Liver When Administered before *CYP1A1* Acrophase

**DOI:** 10.3390/toxics9060130

**Published:** 2021-06-04

**Authors:** Oscar Samuel Ávila-Rosales, Mauricio Díaz-Muñoz, Rafael Camacho-Carranza, Elvia Coballase-Urrutia, José Pedraza-Chaverri, Jorge Omar García-Rebollar, Jesús Javier Espinosa-Aguirre

**Affiliations:** 1 Instituto de Investigaciones Biomédicas, Universidad Nacional Autónoma de México, Ciudad de México 04510, Mexico; x-li@live.com.mx (O.S.Á.-R.); rcamacho@iibiomedicas.unam.mx (R.C.-C.); Jorgerebollar@biomedicas.unam.mx (J.O.G.-R.); 2 Instituto de Neurobiología, Universidad Nacional Autónoma de México, Querétaro 76230, Mexico; mdiaz@comunidad.unam.mx; 3 Laboratorio de Neurociencias, Instituto Nacional de Pediatría, Ciudad de México 04530, Mexico; elcoballase@yahoo.com.mx; 4 Departamento de Biología, Facultad de Química, Universidad Nacional Autónoma de México, Ciudad de México 04510, Mexico; pedraza@unam.mx

**Keywords:** cytochrome P450, benzo[a]pyrene, liver, DNA damage, daytime restricted feeding

## Abstract

The aryl hydrocarbon receptor (AhR) is a ligand-activated transcription factor that heterodimerizes with the AhR nuclear translocator (ARNT) to modulate *CYP1A1* expression, a gene involved in the biotransformation of benzo[a]pyrene (BaP). The AhR pathway shows daily variations under the control of the circadian timing system. Daytime restricted feeding (DRF) entrains the expression of genes involved in the processing of nutrients and xenobiotics to food availability. Therefore, we evaluate if temporal *AhR*, *ARNT,* and *CYP1A1 hepatic* expression in rats are due to light/dark cycles or fasting/feeding cycles promoted by DRF. Our results show that *AhR* oscillates throughout the 24 h period in DRF and ad libitum feeding rats (ALF), showing maximum expression at the same time points. DRF modified the peak of *ARNT* expression at ZT5; meanwhile, ALF animals showed a peak of maximum expression at ZT17. An increased expression of *CYP1A1* was linked to the meal time in both groups of animals. Although a high *CYP1A1* expression has been previously associated with BaP genotoxicity, our results show that, compared with the ALF group, DRF attenuated the BaP-CYP1A1 induction potency, the liver DNA-BaP adducts, the liver concentration of unmetabolized BaP, and the blood aspartate aminotransferase and alanine aminotransferase activities when BaP is administered prior to the acrophase of *CYP1A1* expression. These results demonstrate that DRF modifies the *ARNT* and *CYP1A1* expression and protects from BaP toxicity.

## 1. Introduction

Polycyclic aromatic hydrocarbons (PAHs) are hydrophobic compounds formed and released during incomplete combustion, volcanic eruptions, and diagenesis [1]. The use of petroleum products has increased the concentration of PAHs in the environment [2], and the main sources of exposure to PAHs in humans are cigarette smoke and grilled food [3]. Benzo[a]pyrene (BaP) is considered the prototypical PAH due to its widely studied cytotoxic, mutagenic, and carcinogenic properties [4].

Aryl hydrocarbon receptor (AhR) is a receptor/transcription factor that recognizes BaP, heterodimerizes with AhR nuclear translocator (ARNT), and regulates multiple genes, including members of the cytochrome P450 (CYP) family, such as *CYP1A1* [5]. *CYP1A1* metabolizes BaP into hydrophilic compounds such as 7,8-diol-BaP [6]. However, 7,8-diol-BaP can be metabolized again by *CYP1A1* to yield the highly electrophilic metabolite 7,8-diol-9,10-epoxy-BaP (BPDE) [7] that forms DNA adducts [8], thereby promoting mutagenic and carcinogenic processes [9].

Previous reports indicate that *AhR* expression in rodent liver and lungs show temporal variations [10,11]. These temporal variations are under the control of humoral and neural signals from the suprachiasmatic nucleus (SCN) [12]. Daytime restricted feeding (DRF) is an experimental protocol that limits food access at 2–4 h periods/day during the light period [13]. This fasting/feeding cycle modifies the temporal organization of the liver, kidney, heart, and pancreas activities independently of the SCN [14]. This is achieved through changes in the temporal expression of enzymes involved in the metabolism of lipids, glucose, and urea during the food access period [15,16,17,18].

Additionally, animals exposed to DRF show a decreased toxicity and bioavailability of gentamicin [19], attenuation of acetaminophen toxicity [20], prevention of neoplastic transformation induced by diethylnitrosamine [21], and decreased rates of metastasis when fed with a high-lipid diet [22]. We aimed to determine the effect of DRF on the temporal expression of *AhR*, *ARNT*, and *CYP1A1,* and its influence on BaP-induced hepatotoxicity.

## 2. Materials and Methods

### 2.1. Chemicals

Benzo[a]pyrene, corn oil, moloney murine leukemia virus reverse transcriptase (M-MVL RT), ethylenediaminetetraacetic acid calcium disodium salt (EDTA), DL-dithiothreitol (DTT), glycerol, magnesium chloride (MgCl_2_), ethoxyresorufin-*O*-deethylase (EROD), resorufin sodium salt (RES), dihydronicotinamide adenine dinucleotide phosphate tetrasodium salt (NADPH), ethylene glycol-bis(2-aminoethylether)-*N*,*N*,*N*′,*N*′-tetraacetic acid (EGTA), dimethyl sulfoxide (DMSO), sucrose, sodium dodecyl sulfate (SDS), Proteinase K, and RNase were all purchased from Sigma-Aldrich (St. Louis MO, USA). Potassium chloride (KCl), sodium chloride (NaCl), monobasic potassium phosphate (KH_2_PO_4_), dibasic potassium phosphate (K_2_HPO_4_), phenol, chloroform, isoamyl alcohol, and ethanol were all purchased from JT Baker (Mexico City, Mexico). Trizol was purchased from Invitrogen (Carlsbad, CA, USA). SYBR Green TaqMan Universal PCR Master Mix was acquired from Applied Biosystems (Foster City, CA, USA). Bradford protein assay reagent, Tris Base, polyacrylamide, bis-acrylamide, nitrocellulose membranes, and Tween 20 were all purchased from Bio-Rad Laboratories Inc. (Hercules, CA, USA). Amersham ECL Prime Western Blotting Detection Reagent was acquired from GE Healthcare Life Sciences (Chicago, IL, USA). Mouse monoclonal anti-CYP1A1 (393979) and horseradish peroxidase (HRP)-conjugated secondary antibodies (sc-2354) were acquired from Santa Cruz Biotechnology, Inc. (Dallas, TX, USA). Mouse monoclonal anti-GAPDH (GTX627408) was from GeneTex (Irvine, CA, USA). DNase-free water was acquired from GibcoBRL (Waltham, MA, USA). The BPDER DNA adduct ELISA Kit was acquired from Cell BIOLABS, Inc. (San Diego, CA, USA).

### 2.2. Animals

Adult male Wistar rats, 6 weeks old at the beginning of the experiments, were maintained under a 12:12 h light/dark cycle (ZT0 was lights on and ZT12 lights out) and a constant temperature (22 ± 1 °C), with free access to tap water and food (LabDiet 5001^TM^) for at least one week before the initiation of the experiments. Rats were housed in transparent acrylic cages (40 × 50 × 20 cm) with sani-chips bedding (Envigo^TM^). All experimental procedures were approved and conducted according to the institutional guide for the care and use of animals under biomedical experimentation (Neurobiology Institute, National Autonomous University of Mexico), under the protocol #33 ratified by the Bioethical Committee of Neurobiology Institute (September 2006) and conformed to previously recommended international ethical standards [23].

### 2.3. Animal Treatment

#### 2.3.1. Daytime Restricted Feeding

To examine if the *AhR*, *ARNT*, and *CYP1A1* mRNA acrophase (temporal expression peak) is due to either light/dark cycles or fasting/feeding cycles, we chose the DRF protocol. The animals were placed randomly in one of the following groups: (a) ad libitum feeding (ALF) with free access to water and food, or (b) DRF with access to food from ZT4 to ZT6 for 21 days and free access to water. To determine that the observed effects were neither due to an acute 21 h food-deprivation interval nor to the refeeding after fasting, two additional control groups were included: (c) fasted (Fa), with free access to water and food for 21 days, that were then fasted for 21 h, and (d) rats with free access to water and food for 21 days, that were then fasted for 21 h and refed (Re) for 2 h (from ZT4 to ZT6). Four rats per group (ALF and DRF) were sacrificed by decapitation at ZT0, ZT3, ZT6, ZT9, ZT12, ZT15, ZT18, and ZT21. The Fa and Re rats were sacrificed (four rats per group) at ZT3 (after 21 h fasting) and ZT6 (after 21 h fasting and immediately after 2 h refeeding), respectively.

#### 2.3.2. Exposure to BaP

For genotoxic analysis, the animals were distributed randomly in the ALF or DRF groups. Each group was further divided into three subgroups: (a) the control subgroup, which was sacrificed by decapitation (six rats per group) on the 21st day (DRF group at ZT1 and ALF group at ZT13), or (b) corn oil subgroup, which was intraperitoneally (i.p.) injected on the 21st day with 0.3 mL corn oil at ZT1 (DRF group) and ZT12 (ALF group) and sacrificed by decapitation (six rats per group) at 24 h after administration. Due to the large number of handled animals, this subgroup was included in a preliminary experiment including a control subgroup, (c) the BaP subgroup, which was i.p. injected on the 21st day with 10 mg/kg body weight of BaP in 0.3 mL corn oil at ZT1 (DRF group) and ZT13 (ALF group) and sacrificed by decapitation (six rats per group) at 24, 48, and 72 h after BaP administration. We chose i.p. administration to ensure the absorption of BaP from the peritoneal cavity by the portal system and its subjection to the hepatic first-pass elimination [24].

### 2.4. AhR, ARNT, and CYP1A1 Expression

Total liver RNA was isolated using the TRIzol method [25], and 1 μg of total RNA was reverse-transcribed using M-MVL RT following the manufacturer’s instructions. qPCR for *AhR*, *ARNT*, *CYP1A1,* and *RPS18* (Table 1) was performed with SYBR Green TaqMan Universal PCR Master Mix. Fluorescence was monitored on a CFX96 real-time system (Bio-Rad Laboratories Inc., Hercules, CA, USA), efficiency curves were made for each probe, and the relative expression was calculated using the 2^−ΔΔCt^ mathematical analysis method [26].

### 2.5. Subcellular Fractionation

Three grams of liver were homogenized in 6 mL of 0.15 mM KCl. The homogenate was centrifuged at 9000× *g* for 20 min in a Beckman J2 MC^TM^ centrifuge, and the supernatant was centrifuged at 100,000× *g* for 60 min in a Beckman Coulter^TM^ centrifuge. The pellet was resuspended in 6 mL of buffer 0.1 M K_2_HPO_4_, pH 7.4, and was centrifuged under the same conditions. The pellet was resuspended in 2 mL of microsome buffer 1 mM EDTA, 1 mM DTT, and 20% glycerol.

### 2.6. Protein Quantification

Protein quantification was performed according to Bradford [27], with some modifications in order to allow the acquisition of the results from a microplate. We used 10 µL of a 1:80 dilution of each sample with 200 µL of a 1:4 dilution of the Bradford protein assay reagent. Data were normalized with a bovine serum albumin standard curve.

### 2.7. CYP1A1 Immunodetection

Per lane of hepatic microsomal proteins, 20 µg were separated by 10% SDS-polyacrylamide gel electrophoresis under reducing conditions, transferred to 0.45 µm nitrocellulose membranes, and blocked for 1 h in TBST buffer with 20 mM Tris base, 0.5 M NaCl, and 0.5% Tween 20 pH 7.5. Then, the membrane was incubated overnight at 4 °C with a monoclonal *CYP1A1* antibody or a monoclonal GAPDH antibody, which was diluted 1:2000 (in TBST buffer). After washing, the membranes were incubated with a mouse anti-goat secondary antibody coupled to horseradish peroxidase diluted 1:10,000. The chemiluminescence reaction was performed with an Amersham ECL Prime Western Blotting Detection Reagent. Densitometric analysis was performed using Kodak Image Software (v 3.0).

### 2.8. CYP1A1 Activity Assay

The assay was performed according to the original protocol [28], with some modifications in order to allow the acquisition of the results from a microplate. Forty µg of microsomal protein were incubated at 37 °C for 3 min with 150 µL activity buffer (150 mM Tris base and 25 mM MgCl_2_) and 5 µL of 50 M EROD solution. The reaction was started by adding 40 µL of 5 mM NADPH. The fluorescence assay (520 nm excitation, 585 nm emission) was performed with a Synergy H4^TM^ microplate reader (Biotek Instruments Inc., Winooski, VT, USA). Data were normalized according to the resorufin curve.

### 2.9. BPDE-DNA Assay

For rat liver, 200 µg were homogenized individually in lysis buffer (0.35 M sucrose, 30 mM Tris base, and 2 mM EGTA, pH 7.4) and centrifuged at 12,000× *g* for 10 min at 4 °C in a MIKRO 200R, Hettich^®^. The pellet was resuspended in extraction buffer (10 mM Tris base, 10 mM EDTA, 100 mM NaCl, and 2% SDS, pH 8.0, with 10 µL proteinase K) and incubated at 57 °C for 2 h. Finally, 800 µL of phenol/chloroform/isoamyl alcohol (25:24:1) was mixed with the sample, which was centrifuged at 11,000× *g* for 10 min. The supernatant was collected, and 3 µL of RNase was added. The mixture was incubated for 60 min at room temperature. Then, 500 µL of phenol/chloroform/isoamyl alcohol (25:24:1) was added and the mixture was centrifuged at 11,000× *g* at 4 °C for 10 min. The aqueous phase was collected, and 400 µL of ethanol was added. The sample was centrifuged at 14,000× *g* at 4 °C for 20 min. The supernatant was discarded, and 1 mL of 70% ethanol was added and centrifuged at 14,000× *g* for 10 min, and the pellet was resuspended in DNase-free water. The DNA was quantified in a Nanodrop 2000 (ThermoScientific^TM^). 

We used the BPDE DNA adduct ELISA Kit; briefly, the DNA samples were tested with an anti-BPDE-I antibody, followed by an HRP conjugated secondary antibody, according to the manufacturer’s instructions. The absorbance assay (450 nm) was performed with a Synergy H4^TM^ microplate reader. The BPDE-DNA adduct content in samples was determined by comparing with a standard curve that was prepared from predetermined BPDE-DNA standards [29,30].

### 2.10. Serum Aspartate Aminotransferase (AST) and Alanine Aminotransferase (ALT) Activities

Blood was collected immediately after decapitation and serum was separated by centrifugation at 6000× *g* for 15 min (MIKRO 200R, Hettich^®^), and the supernatant was kept at −80 °C until analysis. AST and ALT activities were determined according to the International Federation of Clinical Chemistry [31] procedure with the Beckman Coulter UniCel^®^ DxC600 Chemistry Analyzer.

### 2.11. BaP Quantification Assay

BaP was measured in 40 µL of rat liver homogenate, 160 µL of DMSO was added, and the fluorescence intensity was measured (470 nm emission, 380 nm excitation) [32] with a Synergy H4^TM^ microplate reader. Data were normalized according to the results for BaP dissolved in DMSO obtained under the same conditions.

### 2.12. Statistical Analysis

The statistical analysis was performed using GraphPad Prism software (v 6.0; San Diego, CA, USA). The normality distribution and equal variances were determined by the Kolmogorov–Smirnov and Levene tests. The results for different time points were compared using a one-way analysis of variance (ANOVA), and the groups were compared using two-way ANOVA; in both cases, ANOVA was followed by a post hoc Bonferroni test. All pairwise multiple comparisons were performed by Student’s *t*-test. Differences among groups were considered statistically significant when *p* ≤ 0.05. For rhythmic interpretation, we considered: acrophase, MESOR, and amplitude calculated with COSANA software (v 3.1 developed by AA Benedito-Silva, GMDRB, ICB/USP, Sao Paulo, Brazil), according to Refenetti et al. [33].

## 3. Results

### 3.1. Liver AhR, ARNT, and CYP1A1 Expression in ALF and DRF Groups

**Figure 1:**
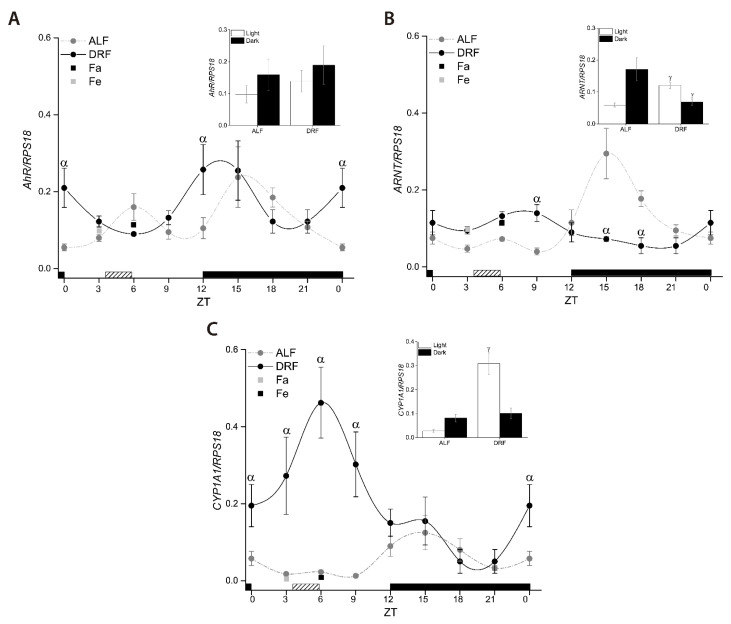
Temporal expression of *AhR* mRNA (**A**), *ARNT* (**B**), and *CYP1A1* mRNA (**C**) in the livers of rats in the ALF (gray circles), DRF (dark circles), Fa (gray squares), and Fe (dark squares) groups. The dark box corresponds to the dark period (ZT12 to ZT0), and the dashed box corresponds to the food access period for the DRF group. Each point represents the mean ± SEM, *n* = 4. α *p* ≤ 0.05 compared to the ALF group at the same time points. γ *p* ≤ 0.05 compared to the ALF group. White bars correspond to the mean ± SEM of all data for the light period, and black bars correspond to the mean ± SEM of all data for the dark period. Points of the same group with significant difference: *AhR* in ALF group: ZT0 vs. ZT15 and ZT0 vs. ZT18; *AhR* in DRF group: ZT6 vs. ZT12 and ZT6 vs. ZT15. *ARNT* in ALF group ZT15 vs. ZT21, ZT15 vs. ZT0, ZT15 vs. ZT3, ZT15 vs. ZT6, and ZT15 vs. ZT9; *ARNT* in DRF group: ZT9 vs. ZT18, ZT9 vs. ZT21, and ZT9 vs. ZT15. *CYP1A1* in ALF group: ZT15 vs. ZT3, ZT15 vs. ZT6, and ZT15 vs. ZT9; *CYP1A1* in DRF group: ZT6 vs. ZT12, ZT6 vs. ZT15, ZT6 vs. ZT18, and ZT6 vs. ZT21.

In rats, under ALF, *AhR*, *ARNT*, and *CYP1A1* mRNA oscillate throughout the 24 h period, showing maximum expression at the interval ZT14 to ZT17 during the dark phase (Figure 1, Table 2). The DRF feeding protocol does not modify the *AhR* peak but suppresses the *ARNT* peak observed in the ALF group at ZT17 (Figure 1A,B, Table 2). It seems that the *ARNT* expression in the animals under DRF is constant throughout the 24 h period, with an expression peak at ZT5 (Figure 1B, Table 2). Surprisingly, in the DRF group, we observed a maximum peak in *CYP1A1* expression at ZT5 that is 15.6-fold higher than that in the ALF group at the same time, but showed the same level of expression at ZT17 (Figure 1C, Table 2). Additionally, no differences were observed in *AhR*, *ARNT,* and *CYP1A1* mRNA levels between the ALF, Fa, or Re groups.

### 3.2. BaP Administration Time

Previous studies associated a high *CYP1A1* expression with an increase in BPDE adducts [8]. In this work, rats under DRF show a high *CYP1A1* expression at mealtime. These results suggests that the period before mealtime in rats under DRF may promote an additive increase in *CYP1A1* induction after BaP exposure as well as an increase in BaP toxicity. To test this hypothesis, we compared the acrophase during both DRF and ALF. Our Cosinor analysis showed that the acrophases were at ZT5 and ZT17 in rats under DRF and ALF, respectively (Table 2). For this reason, we carried out BaP administration 4 h before the acrophase (at ZT1 in the DRF group and at ZT13 in the ALF group), with the purpose of guaranteeing the BaP absorption by the liver [24].

### 3.3. DRF Attenuates the Induction of Hepatic CYP1A1 Expression by BaP

**Figure 2:**
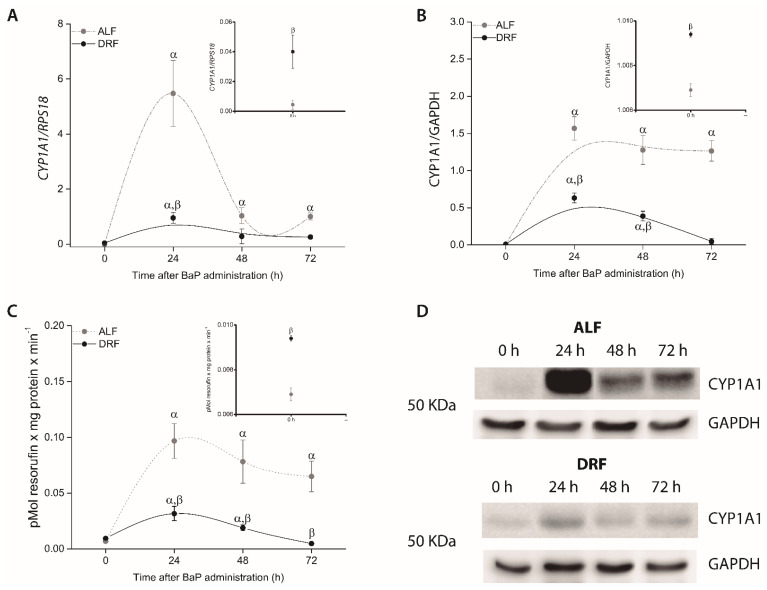
mRNA (**A**), protein levels (**B**,**D**), and activity of *CYP1A1* (**C**) in the livers of rats in the ALF (gray circles) and DRF (black circles) groups after 10 mg/Kg BaP administration. Values correspond to the mean ± SEM, *n* = 6. α *p* ≤ 0.05 compared to the control group with the same diet (DRF at ZT1 and ALF at ZT13) and β *p* ≤ 0.05 compared to the ALF group at the same time points.

In rats under ALF, the maximum *CYP1A1* mRNA expression was observed at 24 h after BaP administration (1237-fold compared with the ALF control group, *p* ≤ 0.05, Figure 2A) and decreased over the subsequent hours. Consistent with the abundance of *CYP1A1* mRNA, its protein level and activity were increased 227- and 14-fold respectively, at 24 h (*p* ≤ 0.05, compared with the ALF control group), and both were maintained until 72 h after BaP exposure (Figure 2B–D).

In the group with DRF, *CYP1A1* mRNA levels were increased 24-fold at 24 h after BaP administration (*p* ≤ 0.05, compared with the DRF control group), but they were 5.72-fold lower than the ALF group at 24 h (*p* ≤ 0.05) and they decreased to the basal expression levels 48 h later. *CYP1A1* protein levels were increased 109- and 41-fold at 24 and 48 h respectively, after BaP administration (*p* ≤ 0.05, compared with the DRF control group), but they were 2.4- and 3.2-fold lower than the ALF group at 24 and 48 h respectively, after BaP administration (*p* ≤ 0.05). *CYP1A1* activity was increased 3.37- and 2.01-fold at 24 and 48 h respectively, after BaP administration (*p* ≤ 0.05, compared with the DRF control group), but it was 3.05- and 4.14-fold lower than the ALF group at 24 and 48 h, respectively (*p* ≤ 0.05). Vehicle control groups (rats under ALF or DRF administrated with only corn oil) did not show a difference between ALF and DRF (Table 3).

### 3.4. DRF Attenuated BPDE-DNA Adduct Levels in the Liver

**Figure 3:**
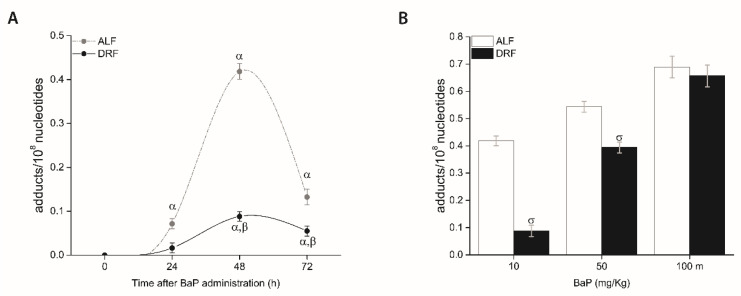
BPDE-DNA adduct levels in the liver of rats with ALF (gray circles) and DRF (black circles) after BaP administration at 10 mg/kg (**A**). BPDE-DNA adduct levels in the liver of rats with ALF (white bar) and DRF (black bar) after BaP administration at 10, 50, or 100 mg/kg (**B**). Each point corresponds to the mean ± SEM, *n* = 6. α *p* ≤ 0.05 compared to the control group that was fed with the same diet (DRF at ZT1 and ALF at ZT13), β *p* ≤ 0.05 compared to the ALF group at the same time point, and σ *p* ≤ 0.05 compared to the ALF group treated with the same concentrations of BaP.

In rats with ALF, the maximum accumulation of BPDE-DNA adducts (0.41843 adducts/10^8^ nucleotides) was observed 48 h after BaP injection (Figure 3). In rats with DRF, BPDE-DNA adduct levels were 0.08821 adducts/10^8^ nucleotides at 48 h, 4.74-fold lower than the ALF group at the same time (*p* ≤ 0.05) (Figure 3A). To determine if the decrease in BPDE-DNA adducts observed at 48 h in the DRF group was also observed at different concentrations of BaP, we evaluated the levels of BPDE-DNA adducts at three different concentrations of BaP (10, 50, and 100 mg/kg). The rats exposed to 50 mg/kg BaP in the DRF group showed decreased (1.4-fold) levels of BPDE-DNA adducts (*p* ≤ 0.05, compared with the ALF group), while the rats exposed to 100 mg/kg BaP did not show a difference. (Figure 3B).

### 3.5. DRF Attenuated Serum AST and ALT Activities after BaP Exposure

**Figure 4:**
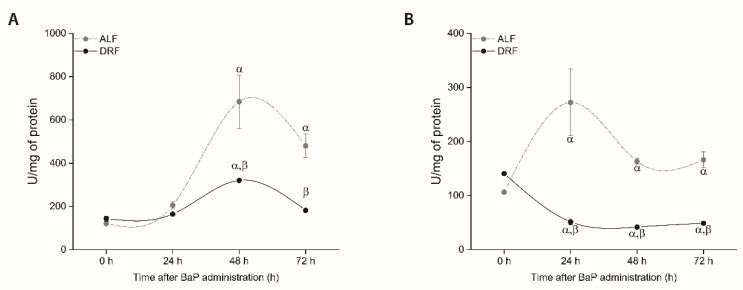
Activities of AST (**A**) and ALT (**B**) in the blood of rats with ALF (gray circles) and DRF (black circles) after BaP administration at 10 mg/kg. Each point corresponds to the mean ± SEM, *n* = 6. α *p* ≤ 0.05 compared to the control group fed with the same diet (DRF at ZT1 and ALF at ZT13) and β *p* ≤ 0.05 compared to the ALF group at the same time points.

In rats under ALF, ALT and AST activities were increased at 24 (2.5-fold) and 48 h (2.13-fold) after BaP exposure respectively, and were maintained higher than ALF control until 72 h (*p* ≤ 0.05) (Figure 4A,B). After BaP injection, rats with DRF qualitatively showed the same pattern in animals under ALF with respect to AST, but with minor intensity (increased 2.13-fold at 48 h, *p* ≤ 0.05, compared with the control group, and decreased at 72 h). Nevertheless, ALT activity in the DRF group decreased 5.5-fold at 24 h and was maintained lower than the control until 72 h (*p* ≤ 0.05) (Figure 4A,B). Vehicle control groups (rats under ALF or DRF administrated only with corn oil) did not show a difference between ALF and DRF (Table 3).

### 3.6. BaP Retention in the Liver

**Figure 5:**
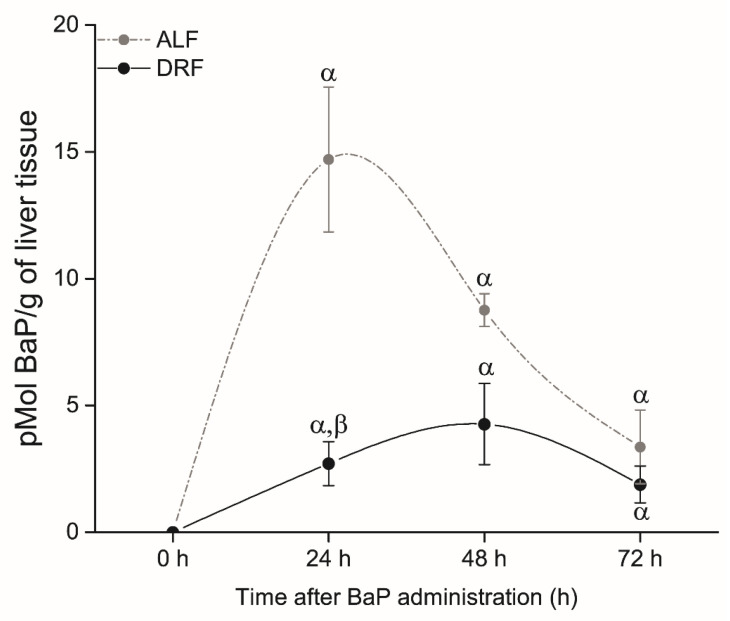
Retention of unmetabolized BaP in the liver of rats with ALF (gray circles) and DRF (black circles) after BaP administration at 10 mg/kg. Each point corresponds to the mean ± SEM, *n* = 6. α *p* ≤ 0.05 compared to the control group fed with the same diet (DRF at ZT1 and ALF at ZT13) and β *p* ≤ 0.05 compared to the ALF group at the same time point.

In rats under ALF, maximal unmetabolized BaP retention (14.7 pMol BaP/g of liver) was observed at 24 h after BaP administration, and decreased at subsequent times recorded (Figure 5). In rats with DRF, we observed a 5.4-fold decrease in unmetabolized BaP retention at 24 h (*p* ≤ 0.05, compared to the ALF group at the same time point), which decreased at subsequent times recorded (Figure 5). It is worthy to note that the liver concentration of total unmetabolized BaP at 24 h was lower in the DRF group than in the ALF group of animals.

## 4. Discussion

In rats under ALF, the SCN controls behavioral and physiological rhythms. Nevertheless, when the food availability is restricted to a specific and shorter-than-usual time at a particular time each day (DRF protocol), rats show a second period of arousal and increased locomotor activity that occurs before the time of the daily mealtime (called food-anticipatory activity), which is independent of signaling by the SCN [34]. Moreover, in the liver, clock genes such as *Bmal1*, *Per1*, and *Per2* modify their temporal pattern [35,36], and enzymes involved in nutrients and xenobiotics metabolism are modified according to the mealtime [34].

The genes modulated by the clock genes have one or more consensus-binding sites for the molecular clock (E-box) in their promoter region [37]. The *AhR* promoter has an E-box^11^, and this could be responsible for its temporal variation observed in both the ALF and DRF groups (Figure 1A), and in previous reports by others [10]. One peak of maximal expression was observed in both groups of rats at the same time (ZT14). The *AhR* promoter contains a glucocorticoid-responsive element, as well as others such as cAMP-, antioxidant-, and dioxin-responsive elements [38,39], suggesting that the *AhR* expression depends on more than one factor to promote its transcription.

In rats under ALF, *ARNT* shows a peak of higher expression at ZT17 (Figure 1B), and this could be due to the positive modulation by albumin D-element binding protein (DBP) that shows temporal variation with a peak in the early night [40]. During the restricted feeding protocol, the temporal pattern in DBP is modified in the liver, increasing during the light phase (mealtime) but decreasing in the night phase [41], as *ARNT* expression is BDP-dependent, and this could be a possible factor involved in the almost similar expression of *ARNT* throughout the 24 h time period in DRF rats (Figure 1B).

*CYP1A1* has shown variations during a 24 h period in the liver, lung, and SCN [11,42] that coincide with the period of highest food consumption [23]. Through food, ingested compounds may activate the AhR pathway, inducing *CYP1A1* expression [43], suggesting that *CYP1A1* increase could be related to food access. Therefore, we modified food access to 2 h per day in the light period (ZT4 to ZT6) for 21 days, promoting an increase in *CYP1A1* expression before the mealtime. These results suggest that modification of *CYP1A1* expression is associated with constant fasting/feeding cycles, and there are at least two possible explanations for these data. First, an endogenous ligand that increases during the time before mealtime may activate AhR, leading to *CYP1A1* mRNA induction, where multiple potential ligands to AhR such as tryptophan metabolites, heme metabolites, and arachidonic acid metabolites have been proposed [43]. Second, a non-canonical mechanism mediated by kinase activity induces *CYP1A1* [44].

Previous reports have shown that rats with acute-fasting increased IL-1α, IL-6, and TNF-α [36], enzymes involved in gluconeogenesis (G6pasa PEPCK) [17], ketonic bodies, and sirtuin [16]. However, our results showed that *AhR*, *ARNT*, and *CYP1A1* expression were not modified during acute-fasting (Fa group) or acute-fasting and 2 h refeeding (Re group). These results suggest that the observed effect in these genes is due to constant fasting/refeeding cycles.

PAHs have been classified as human carcinogens by the International Agency for Research on Cancer (IARC) [45]. BaP is the most studied PAH and is considered a model compound that can be metabolized by *CYP1A1*, contributing to 48.3% of BPDE, the ultimate reactive species that can react with DNA, promoting mutagenic and carcinogenic processes. Additionally, other CYPs, such as CYP1B1, CYP3A4, and CYP2C19 [46], and cytosolic enzymes such as 5-lipoxygenase or cyclooxygenase 1/2, are able to participate to a lesser extent than *CYP1A1* in the bioactivation of BaP [47]. 

In regard to the *CYP1A1* induction property of BaP, *CYP1A1* expression and protein concentration and activity in the DRF group were lower than that observed in the ALF group (Figure 2). As reported by others [48,49,50,51], the single i.p. administration of BaP to rats with free access to water and food produced the maximal levels of BPDE-DNA adducts two days after administration, and they decayed at 72 h. In the case of rats under DRF, at 48 h after BaP administration, the levels of BPDE-DNA adducts were lower than those found in animals under ALF (Figure 3A). This protective effect is also observed in rats under DRF that received 50 mg/kg BaP compared with rats under ALF (Figure 3), which coincided with a low *CYP1A1* activity in rats under DRF (data not shown). 

BaP toxicity is not limited to DNA damage since it was associated to an increased serum AST and ALT, suggesting acute liver necrotic activity [52,53]. We found that AST activity was less increased in the DRF group compared to ALF, and ALT activity was decreased (Figure 4). These results may also be related to a decrease in the metabolism of BaP due to a less increased induction and activity of *CYP1A1* after BaP administration (Figure 2). This assumption is supported by the fact that the protective effect of DRF against genotoxicity is minimized in a dose-related way if the used BaP concentrations are increased (Figure 3B).

DRF is a protocol that includes two features: (a) a decreased caloric intake and (b) periodicity in food availability [54]. Previous reports show that a low caloric intake contributes to longevity and multiple health benefits [55], however, it increases basal *CYP1A1* activity (but with no changes in its temporal pattern) [56], promoting increases in BPDE-DNA adducts [57]. The differences between the above findings and our results are evidence to support that DRF and caloric restriction regulate different metabolic pathways [54].

We cannot rule out that there may be additional mechanisms involved in DRF-mediated protection. Previous reports show that DRF modifies the valproic acid concentration, absorption, and pharmacokinetics during mealtime [58,59,60], and decreases gentamicin bioaccumulation [19]. Due to the heterogeneity in the chemical structure of the above-mentioned xenobiotics, we suggest that a process associated with fasting/feeding cycles may be involved. A possible candidate process is blood flow, since it was observed that it influences drug absorption, metabolism, excretion, and blood flow to the enteric system increases during mealtime [60,61,62,63]. Additionally, DRF modifies renal excretion [64]. An alternative/additional hypothesis is that during DRF, heart rate and blood pressure show an acute increase in the period before food access, favoring mobilization of BaP (and other xenobiotics) outside the liver and promoting their excretion, as observed in the case of methotrexate [65]. Concerning this matter, we found that DRF decreases BaP retention in the liver, which was correlated with low liver *CYP1A1* levels and BPDE-DNA adducts as well as diminished transaminases activity in the blood. These results suggest that the reduction of BaP retention during DRF could be an additional factor in the attenuation of BaP liver genotoxicity.

We do not discard that some endogenous, weak AhR ligands (indigoids, heme metabolites, eicosanoid, or equilenin) [66] could interfere with the CYP-induction properties of BaP, as observed with natural compounds such as quercetin or kaempferol [67]. 

In this study, we presented evidence that DRF increases *CYP1A1* expression during the time before food intake but decreases the *CYP1A1* induction potency of BaP when the xenobiotic is administered before the *CYP1A1* acrophase. Additionally, rats under DRF are resistant to liver toxicity by BaP as BPDE-DNA adducts and AST and ALT activities are lower than those observed in ALF rats. The time-lag between *AhR* and *ARNT* acrophase caused by DRF may be partly responsible for these results, although additional physiological factors leading to poor liver retention of unmetabolized BaP could also be involved. Finally, we cannot exclude the possibility that BaP administration at different times in the ALF and DRF groups may influence the obtained results.

## Figures and Tables

**Table 1:** Primer sequences for amplification of rat *AhR*, *ARNT*, *CYP1A1,* and *RPS18* by real-time PCR.

Gene	NCBI RefSeq	Forward	Reverse
*AhR*	NM_013149.3	5′-gggccaagagcttctttgatg-3′	5′-gcaagtcctgccagtctctga-3′
*ARNT*	NM_012780	5′-agagacttgccagggaaaatcata-3′	5′-tttcgagccagggcactacagg-3′
*CYP1A1*	NM_012540.2	5′-gggccaagagcttctttgatg-3′	5′-gtcccggatgrggcccttctcaaa-3′
*RPS18*	NM_213557.1	5′-ttcagcacatcctgcgagta-3′	5′-ttggtgaggtcaatgtctgc-3′

**Table 2:** Chronobiological parameters obtained by Cosinor analysis for the effects of DRF on *AhR*, *ARNT,* and *CYP1A1* expression.

	Mesor		Amplitude		Acrophase	
	ALF	DRF	ALF	DRF	ALF	DRF
*AhR*	0.128	0.164	0.091	0.084	ZT14	ZT14
	±0.014	±0.017	±0.026	±0.027	±1.936	±2.44
*ARNT*	0.115	0.094	0.128	0.043	ZT17	ZT5
	±0.02	±0.047	±0.03	±0.007 ^α^	±0.75	±1.43 ^α^
*CYP1A1*	0.055	0.205	0.056	0.207	ZT17	ZT5
	±0.009	±0.031 ^α^	±0.018	±0.041 ^α^	±1.436	±3.86 ^α^

Each data point corresponds to the mean + SEM, *n* = 4. ^α^ *p* ≤ 0.05 compared to the ALF group.

**Table 3:** Effect of vehicle (corn oil) administrated i.p. in rats during ALF or DRF protocol on the expression/activity of *CYP1A1*, and transaminase serum activities.

	Control		Only Corn Oil	
	ALF	DRF	ALF	DRF
*CYP1A1*/RPS18	0.0043 ± 0.0003	0.0400 ± 0.002	0.0046 ± 0.0010	0.0394 ± 0.005
*CYP1A1* activity (pMol resorufin × mg protein × min^−1^)	0.0069 ± 0.0002	0.0094 ± 0.0001	0.0067 ± 0.0001	0.0096 ± 0.0001
ALT U/mg protein	106.3 ± 1.91	141 ± 2.8	106.7 ± 2.61	140 ± 5.8
AST U/mg protein	121.5 ± 5.3	143.5 ± 10.6	125.5 ± 7.2	145.2 ± 9.6

Each data point corresponds to the mean ± SEM, *n* = 6.

## Data Availability

Data is contained within the article.
